# Telomere length associated with the risks of high-risk and ischemic stroke in southern Chinese Han population

**DOI:** 10.18632/oncotarget.22509

**Published:** 2017-11-18

**Authors:** Dong Luo, Qun Hou, Jianzhong Yu, Dan Yu

**Affiliations:** ^1^ Department of Neurology, School of Medicine, The Second Affiliated Hospital of Zhejiang University, Hangzhou 310009, Zhejiang, China; ^2^ Department of Neurology, Zhejiang Hospital of Traditional Chinese Medicine, Hangzhou 310006, Zhejiang, China; ^3^ Department of Neurology, Affiliated Haikou Hospital of Xiangya Medical College of Central South University, Haikou 570208, Hainan, China

**Keywords:** telomere length, ischemic stroke, high-risk stroke population

## Abstract

Some previous studies suggested telomere length was associated with the risk of ischemic stroke (IS). The aim of this study was to further confirm the association between relative telomere length (RTL) and risk of IS and to especially explore its correlation with the risk of high-risk stroke population in southern Chinese Han. RTL was determined by using real-time quantitative polymerase chain reaction from 400 ischemic stroke patients, 409 high-risk stroke populations and 399 healthy controls. The correlations between the controls and the risk of high-risk and ischemic stroke were evaluated by using an unconditional logistic regression. IS patients have shown longer RTL than controls (median1.52vs1.11, p60 years) and gender suggested that the first and second tertile of RTL were correlated with the risk of IS in each group when the second tertile was used as a reference. However, the increased risk for high-risk stroke populations were only presented in the first tertile of RTL in the age≤60 years and female groups. the RTL was associated with an increased risk of ischemic stroke, while it elevated the risk of high-risk stroke in some specific subpopulations.

## INTRODUCTION

Stroke is a global health problem and is the second commonest cause of death and a leading cause of adult disability worldwide [1, 2]. And ischemic stroke as the most common type of stroke accounts for 43.7%-79.9% of all strokes [3, 4]. A recent Chinese study has shown that the annual mortality from stroke was about 157 per 100000 which exceedsheart disease, becoming the leading cause of death and adult disability [4]. Due to the huge economic and social burden caused by stroke, it is vital to identify the biomarkers aiding in both prediction and prevention of this disease, excluding the traditional risk factors.

Telomeres are the end DNA-protein capping structure maintaining the eukaryotic genomic integrity and stability by preventing end-to-end fusion between chromosomes [5]. Telomeres ends become shorter with each cell division, which is associated with cellular senescence and apoptosis [6]. The process can be further accelerated with the exposure to severe chronic inflammation and oxidative stress [7, 8]. Cellular senescence parallels the development of atherosclerosis and other pathologies in the vasculature; therefore, it is likely to have a core role in cardiovascular disease [9]. In addition, as telomere length within individuals is generally strongly correlated across tissue types, leucocyte based measurement might also equate with telomere length in less accessible tissues [10]. Thus leucocyte telomere length (LTL) can be considered a predictor of vascular ageing, and further indicate the prediction of atherosclerosis and ischemic stroke.

The research on the association between telomere length and chronic diseases has increased recently. Evidences have demonstrated that shorter leucocyte telomere length was related with a higher risk of coronary heart disease [11] and had been confirmed in two meta-analyses [12, 13]. However, as for ischemic stroke, these two meta-analyses showed opposite correlation with shorter LTL. Most of the previous studies about LTL are limited to the linkage between stroke patients and healthy controls [14, 15] or on patients only [16], with less information on the high-risk stroke populations. The high-risk populations, as an intermediate status between the healthy and ischemic stroke, are a priority to primary prevention from stroke. Thus, it is interesting for us to test whether LTL can be used as a biomarker for identification of the high-risk stroke population. Therefore, we designed this case - high risk - control study, which was to clarify whether LTL was associated with high-risk population and ischemic stroke in southern Chinese Han populations.

## RESULTS

### Demographic and clinical characteristics

A total of 1208 samples (400 cases, 409 high-risk subjects, 399 controls) were included in the present study. The baseline characteristics of the three groups (case, control, high-risk) were shown in Table 1. There were significant differences in age, gender, total cholesterol (TC), total triglyceride(TG), high density lipoprotein-cholesterol (HDL), low density lipoprotein-cholesterol (LDL) between the high-risk and control group. The statistic differences were detected in age, TC, TG, HDL between the case and control group but not in gender and LDL.

**Table 1:** Clinical and epidemiological characteristics of the high-risk and case group compared with the control group

	Control	High-risk	Case	P_1_	P_2_
Age	48.66±11.07	65.31±11.45	66.84±11.65	<0.001	<0.001
Gender					
male(%)	61.7	43	65.8	<0.001	0.052
TC	5.22±0.86	2.16±1.26	4.92±1.24	<0.001	<0.001
TG	1.51±0.54	5.73±1.12	1.34±0.94	<0.001	0.012
HDL	1.34±0.51	1.50±0.46	1.27±0.33	<0.001	0.027
LDL	3.17±0.72	3.68±0.92	3.00±1.78	<0.001	0.053

P_1_ P_2_ compare between the high-risk, case and control group respectively; total cholesterol (TC); total triglyceride(TG); high density lipoprotein-cholesterol (HDL); low density lipoprotein-cholesterol (LDL).

### Correlations between RTL and age

**Figure 1:**
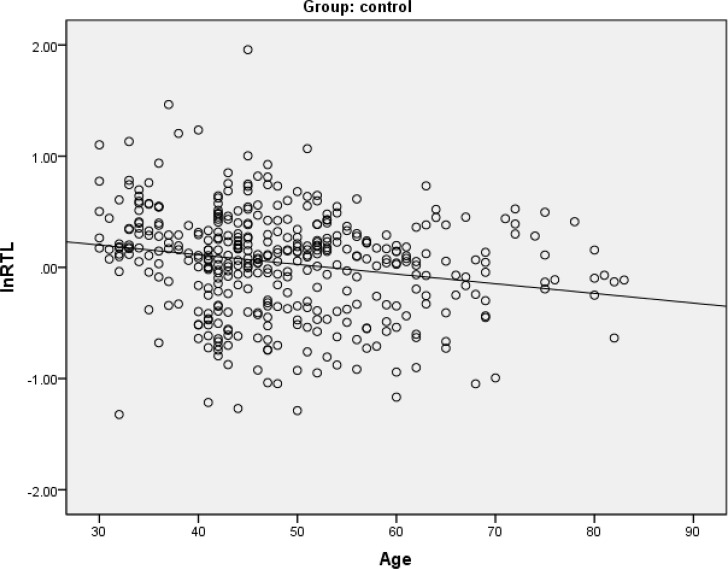
Spearman's correlation analysis between relative telomere length and age in healthy controls There were negative correlations between relative telomere length (RTL) and age in this group.

We performed spearman's correlation analysis to explore the relation between RTL and age among these three groups. The results showed that RTL was negative correlation with age in the control group (Figure 1), with a correlation coefficient (ρ) of − 0.22 (P<0.001). A negative trend between RTL and age were found in the case group (ρ= − 0.10, p=0.052). There was no significant difference observed in the high-risk group.

### The RTL analysis in the high-risk and ischemic stroke

As shown in Table 2, the median and quartile range (QR) were 1.11(0.77-1.39) in the controls, 1.05(0.48-1.41) in the high-risk stroke group and 1.52(0.47-1.93) in the case group. RTL in the controls was notably shorter than that in the cases, while it was longer than that in the high-risk group. Significant differences were observed in total RTL and each subgroups (female, male, age ≤60 years, age >60 years) among the three groups. However, there was no statistical significance between the control and high-risk stroke group in the male and age > 60 years groups.

**Table 2:** Distribution of relative telomere length among the total, gender and age groups in all participants

		Control			High-risk1			Case	p			
		N	M(QR)	N	M(QR)	N	M(QR)	co-hr-ca	co-hr	co-ca	ca-hr
Total		399	1.11(0.77-1.39)	409	1.05(0.48-1.41)	400	1.52(0.47-1.93)	<0.001	0.027	<0.001	<0.001
Gender	Male	246	1.09(0.71-1.36)	176	1.09(0.77-1.45)	263	1.60(0.56-1.96)	<0.001	0.905	<0.001	<0.001
	Female	153	1.16(0.87-1.46)	233	1.01(0.36-1.40)	137	1.37(0.38-1.86)	0.001	0.002	0.341	0.002
	p		0.101		0.096		0.044				
Age	≤60 years	340	1.14(0.75-1.40)	137	1.04(0.42-1.38)	110	1.66(0.66-2.05)	<0.001	0.02	<0.001	<0.001
	> 60 years	59	0.93(0.78-1.20)	272	1.05(0.58-1.44)	290	1.46(0.45-1.86)	<0.001	0.44	0.005	<0.001
	p		0.024		0.662		0.035				

M(QR), median (quartile range); co, control; ca, case; hr, high-risk.

To assess the associations between the three groups, we performed an unconditional multivariate regression analysis adjusted for age and sex. The samples were divided into three groups based on the tertile values of RTL in controls. When the second tertile was considered as reference, the ORs (95%CI) in the first and third tertile of RTL were 2.88(1.88-4.41) and 6.62(4.32-10.15) in the case group, which exhibited a nonlinear relation between the RTL and the ischemic stroke risk. However, as for the high-risk group, no statistical significances were found in the first (OR 1.28, 95%CI 0.89-1.84) and third tertile (OR 1.23,95%CI 0.82-1.82).

**Figure 2:**
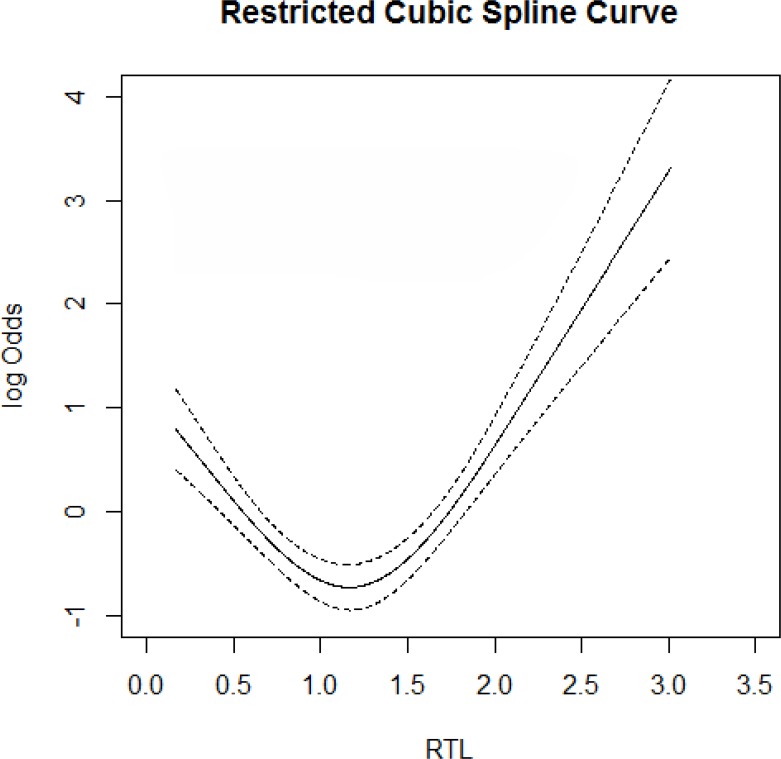
RTL on the risk of ischemic stroke A U-shaped relationship was observed among them. solid line, OR; dotted line, 95%CI.

**Figure 3:**
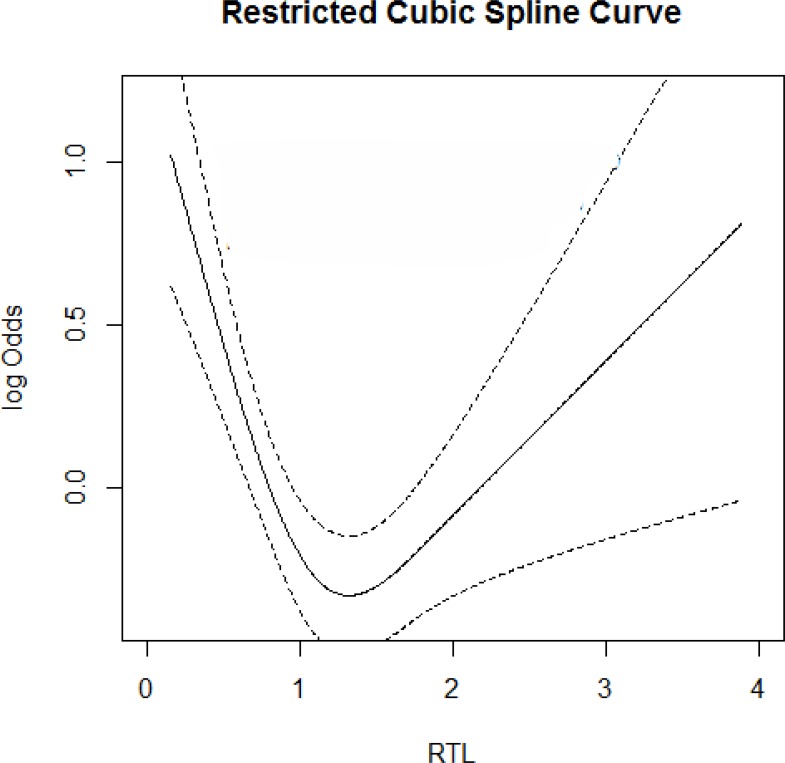
RTL on the risk of high-risk population A U-shaped relationship was observed among them. solid line, OR; dotted line, 95%CI.

We further performed a restricted cubic spine curve to express the correlation between RTL and the risk of IS and high-risk stroke. As shown in Figure 2, The results presented a typical U-shaped association between the risk of IS and the RTL, which meant that either shorter or longer RTL was associated with increased risk of IS. In the high-risk group compared with the controls, this U-shaped correlation still existed (Figure 3).

To further exclude the confounding effects of age and gender, we conducted a stratified analysis and found that they had no impact on the association between RTL and the risk of IS (Table 3). Either shorter or longer RTL was associated with the risk of ischemic stroke among each age or gender group. As for the high-risk samples, the significant differences was only observed in first tertile of RTL in the female and age≤60 years groups.

**Table 3:** Association between relative telomere length and high-risk and ischemic stroke risk stratified by age and gender

		High-risk		Case	
		OR(95%CI)	p	OR(95%CI)	p
Age≤60	≤0.89	1.80(1.14-2.87)	0.013	2.41(1.28-4.54)	0.006
	0.89-1.43	1		1	
	≥1.43	1.21(0.71-2.05)	0.483	6.42(3.59-11.49)	<0.001
Age>60	≤0.89	0.84(0.44-1.60)	0.6	2.28(1.16-4.48)	0.017
	0.89-1.43	1		1	
	≥1.43	1.26(0.59-2.70)	0.55	6.78(3.14-14.65)	<0.001
Male	≤0.89	0.92(0.56-1.53)	0.757	2.56(1.50-4.39)	0.001
	0.89-1.43	1		1	
	≥1.43	1.29(0.75-2.21)	0.365	8.01(4.67-13.76)	<0.001
Female	≤0.89	1.96(1.14-3.40)	0.016	3.43(1.70-6.89)	0.001
	0.89-1.43	1			
	≥1.43	1.14(0.64-2.04)	0.663	4.78(2.39-9.57)	<0.001

P, the control group was compared.

## DISCUSSION

In this study, we examined the RTL in peripheral blood leukocytes from the control, high-risk and ischemic stroke samples to evaluate the association between them. The results suggested that RTL was notably longer in the IS than in the control or high-risk group, while it was shorter in the high-risk samples than in controls. Most importantly, logistic regression analysis suggested that either shorter or longer RTL could increase the risk of IS, indicating U-shaped association between RTL and IS risk. Though no significant difference was found between the control and high-risk group in logistic regression analysis, the results indicated there was a U-shaped association between them.

To date, numerous studies have been conducted to examine the associations between the relative telomere length and the risk of IS. However, the conclusions are inconsistent, some studies demonstrated that shortened RTL was significantly associated with the risk of stroke [15, 17, 18], while meta-analysis and studies indicated shorter telomeres were not correlated with the risk of cerebrovascular disease, as shown in studies with a high quality score or in prospective studies [13, 19, 20]. Interestingly, our results showed that RTL was obviously longer in the case group than in the controls, and also increased the risk of IS, which was less reported in ischemic stroke before. Previous studies suggested longer RTL was mainly reported to be associated with increased risks of some cancers such as lung [21], skin melanoma [22], breast [23] cancers. However, some other studies demonstrated that shorter RTL was also associated with increased risks of lung [24], breast [25] and stomach cancers [26]. The studies on cancers have shown dual roles of telomere in the development of diseases, which may also exist in ischemic stroke study. In our study, the results of RTL by tertile suggested that the first and third tertile were significantly different from the second after adjustment for age and gender, indicating either longer or shorter RTL increased the risk of IS. The discrepancies between longer and shorter RTL in cases than in controls may be partially explained by differences of race and region, timing of sample collection or confounding risk factors. Therefore, large studies, particularly in prospective settings, are necessary to confirm these findings.

Until now, for the ischemic stroke, the thrombolysis and endovascular therapy are the most effective ways to reduce the rate of disability and recover the brain function, but they can't be widely used for limitation of the narrow therapeutic window. Therefore, preventive treatment for the high-risk stroke populations can receive twice the results with half the effort. However, according to the inclusion criteria, high-risk stroke people screened may not be the real one because of some uncertain risk factors within the inclusion criteria such as lipid level, stroke status or physical activity which is hard to be defined correctly. Then for the purpose to identify real high-risk populations, we conducted this study to evaluate the representative of RTL as a biomarker for the high-risk stroke populations. The results suggested that RTL was significantly associated with the risk of high-risk populations. To confirm this association, the stratified analysis was performed based on the tertile grouping of RTL in the controls. However, the regression analyses indicated that the first and third tertile of RTL were not associated with the high-risk populations when using the second tertile as reference. Further analysis performed on the subgroup of age and gender, the results showed that the first tertile was associated with an increased risk of high-risk stroke populations in female and age ≤60 years groups. The conclusion may be partially related with the enrollment risk factors of the high-risk group. After all, the associations between RTL and the risk of hypertension, diabetes, blood lipid were unconfirmed [27–30].

In this present study, some limitations should be noted. Selection bias of the high-risk and ischemic stroke populations cannot be avoided, such as the age selection in the high-risk group and the mismatch of gender and age among some groups. In addition, some other information such as medicine usage, cigarette smoking, and physical activity were not collected. For example, some previous study showed that statins usage were associated with the modulation of EPC cellular senescence [31].

In summary, the study made a preliminary exploration on the associations between RTL and the risk of high-risk and ischemic stroke in southern Han Chinese. The results indicated that relative telomere length was associated with an elevated risk of ischemic stroke, and also increased the risk of high-risk stroke in some specific subpopulations. However, more replication researches of RTL were needed to confirm the clinical utility for ischemic stroke, especially for the high-risk stroke populations. If these findings were confirmed, RTL may serve as an informative marker to improve the prevention and treatment of ischemic stroke.

## MATERIALS AND METHODS

A total of 400 ischemic stroke patients were consecutively recruited when attending the Neurology department of Affiliated Haikou Hospital of Xiangya Medical College of Central South University, China, from February 2015 to March 2016. The diagnosis was defined as the sudden onset of focal neurologic deficit for over 24 hours, confirmed by combining physical examination and radiological test (computed tomography scan and/or magnetic resonance imaging) in strict accordance with the International Classification of Diseases (9th Revision). The inclusion was patients with initial acute atherosclerotic cerebral infarction. We excluded other subtypes of ischemic stroke (small-artery occlusion lacunar, cardioembolism, undetermined stroke) and system diseases (collagenosis, inflammation, liver or renal diseases).

Screening for the high-risk samples were conducted in Hainan province from 2014, according to the guidelines for stroke screening and prevention published by the Ministry of Health in China. All of the 409 high-risk samples consisted of those with age over 40 years who had lived for at least five years locally. High-risk subjects must satisfy at least three of the seven following conditions including hypertension (blood pressure ≥ 140/90mmHg, and/or having received treatment for hypertension), atrial fibrillation or valvular heart disease, diabetes mellitus, physical inactivity(physical activity are recognized by at least 40 minutes per day, 3 to 4 days per week over one year, or participation in industrial and agricultural manual work), dyslipidemia or unknown, obvious overweight or obesity(body weight index BMI ≥ 26Kg/m^2^).

The 399 controls were recruited from the health checkup center in our own hospital. We excluded the subjects with history of cerebrovascular and cardiovascular disease (e.g., myocardial infarction and coronary artery disease) as well as subjects with autoimmune diseases. The demographic and clinical data were collected from each participant by inquiry, questionnaire or medical records. All participants were genetically unrelated Han Chinese and local residents of Hainan province.

The study was approved by the Clinical Research Ethics of Haikou Municipal Hospital. Written informed consent was obtained from all the subjects participating in the present study.

### Relative telomere length measurement

Genomic DNA were extracted from peripheral venous blood of all participants by the GoldMag-Mini Purification Kit (GoldMag Co. Ltd. Xian city, China) according to manufacturer's instructions. All the DNA samples were accurately quantified by the NanoDrop 2000 (Thermo Scientific, Waltham, Massachusetts, USA). Real-time quantitative PCR (RT-PCR)-based method was used to calculate the RTL in a LightCycler®480 QPCR System (Roche, Basel, Switzerland). RTL was the ratio between the copy number of telomere repeats (T) and a single-copy gene copy number (S). The derived T/S ratio for each sample was normalized to that of a calibrator DNA sample to standardize differences between runs and the normalized T/S ratio was defined as RTL.

### Statistical analysis

All statistical analyses were performed on the IBM SPSS Statistics 19.0 software (IBM). Continuous data were expressed as mean + standard deviation (SD), while categorical variables were shown as percent. Differences of categorical variables were assessed by the chi-square test (χ2). Quantitative data was statistically analyzed with analysis of variance (ANOVA) and post hoc tests. Spearman correlation analysis was conducted to explore the relationship between RTL and age. The association between RTL and the risk of high-risk and ischemic stroke was estimated by odds ratio (OR) and 95% confidence interval (95%CI) using an unconditional multivariate logistic regression model with adjustment for age and sex. A restricted cubic spline curve was drawn in the logistic regression model to assess the potential association. All statistical tests were two-sided, and statistical significance was set at p<0.05.
